# Comparative Evaluation of Structured Clinical Case Examination Versus Traditional Long Case Examination for Assessing Clinical Competence in Pediatric Patients in India: A Quasi-experimental Study

**DOI:** 10.7759/cureus.98226

**Published:** 2025-12-01

**Authors:** Vinayaka Gopal, Niranjan Kamble, Vijayasuryakiran KM, Niveditha Hegde Venkatramana, Darshan Rajatadri Rangaswamy

**Affiliations:** 1 Department of Pediatrics, Subbaiah Institute of Medical Sciences and Research Center, Shivamogga, IND

**Keywords:** clinical competence, evaluation studies, mbbs, medical education, undergraduate education

## Abstract

Purpose: Due to subjectivity, traditional long cases present challenges in assessing clinical acumen. Implementing the Objective Structured Long Examination Record to tackle these concerns presents practical challenges. These challenges lead to considering the Structured Clinical Case Examination (SCCE) as a middle ground. This article aims to answer how clinical competence assessment in pediatrics differs when using a Structured Clinical Long Case Examination rather than a Traditional Long Case Examination (TLCE).

Methods: This study was conducted at the Subbaiah Institute of Medical Sciences, Shivamogga. The study focused on 133 final-year MBBS undergraduates during their pediatric posting. Students were assessed using TLCE and SCCE by two different faculties on the same day using a validated checklist for SCCE. The students’ perceptions were collected via a questionnaire. The paired sample t-test compared TLCE and SCCE scores for a comprehensive evaluation.

Results: The paired sample t-test revealed a significant increase in marks obtained in SCCE (mean = 29.8, standard deviation, SD = 5.4) compared to TLCE (mean = 26.7, SD = 5.9) (t = 7.21, p < 0.001). Notably, 84.2% of students passed SCCE, compared to 69.9% passing TLCE. A higher proportion of students scored good (31.5%) and excellent (21.8%) marks in SCCE compared to TLCE (18.7% for good, 10.5% for excellent). Feedback from a Likert-scale questionnaire indicated strong student agreement with SCCE (χ² = 13.7, p = 0.008).

Conclusion: SCCE appears to be a useful structured method for assessing the clinical competency of final-year medical students, offering a more organized evaluation of diverse clinical skills. Students reported favorable perceptions of SCCE, particularly regarding clarity of expectations and inclusion of communication skills. In conclusion, SCCE's structured, observational approach has the potential to address inadequacies in clinical evaluation and enhance clinical skill learning in graduates. Further studies are needed to confirm its broader applicability and effectiveness.

## Introduction

Assessment is pivotal in certifying medical practitioners and driving learning within the medical education process. Long- and short-case exams have been conventionally employed to evaluate clinical skills due to their emulation of real-world scenarios, garnering appreciation for their authenticity [1]. However, persistent challenges have been documented over time, including low validity, subjectivity, examiner bias, and lack of generalizability. Additionally, concerns raised by students regarding time constraints, uncooperative patients, and language barriers underscore the need for standardized assessment systems to enhance the quality of evaluations [2,3].

The traditional MBBS long-case, while widely utilized, has faced criticism for its limited validity, reliability, and inability to assess candidates across a broad spectrum of clinical situations and skills. This format's reliance on subjective grading and the potential for examiner-induced variability further compound its shortcomings [3].

Specifically, reliability concerns have been highlighted in long-case clinical evaluations, where students engage with patients without supervision for approximately an hour before presenting their findings to examiners. Despite its use in assessing clinical skills later in training, the long-case format lacks dependability [4].

Various modifications have been proposed to address these limitations, such as direct observation, extended testing times, increased examiner numbers, and structured marking grids [2]. However, a definitive solution to enhance the reliability and validity of clinical assessments remains elusive. Traditional long case examination (TLCE) relies on unsupervised patient interaction followed by holistic presentation with global rating by the examiner, while the Structured Clinical Case Examination (SCCE) uses structured direct observation with itemized scoring for each component based on a validated checklist.

The SCCE emerges as a structured assessment technique designed to mitigate the shortcomings of traditional long-case exams [2]. SCCE offers an impartial and standardized evaluation of clinical abilities and competencies by reducing examiner anxiety and eliminating marking variability. Its demonstrated efficacy in improving the validity and reliability of clinical assessments has propelled its widespread adoption in medical education [3,4]. Hence, we conducted this study to determine how clinical competence assessment in pediatrics differs when using a Structured Clinical Long Case Examination vs. a TLCE.

## Materials and methods

Objectives

This study aimed to investigate how the clinical competence assessment in pediatrics differs when using a Structured Clinical Long Case Examination compared to a TLCE. The primary aim is to compare the scores of the SCCE assessment method with the traditional long-case examination (TLCE), and the secondary objective is to study the perception of this new method among students.

Study design and setting

This study is quasi-experimental, utilizing a single-group design with two assessment points for comparison. The study was conducted at the Department of Pediatrics in the Subbaiah Institute of Medical Sciences, Shimoga. The study lasted four months, from December 2021 to March 2022. The data were collected and stored in an Excel spreadsheet (Microsoft Corporation, Redmond, WA). Final-year MBBS students who completed a Pediatrics clinical rotation were included if they met the following criteria.

The study included all final-year MBBS students who attended the assessment and excluded students who were absent on the day of the assessment.

Ethics statement

The study was initiated after obtaining approval from the Ethics Committee of the Subbaiah Institute of Medical Sciences, with IEC Code SUIMS/R&D/IEC/074/2021.

Interventions

The study involved implementing a new examination method called SCCE, which was introduced to students through classroom sessions where they were briefed about the new assessment format and encouraged to ask questions for clarity. The SCCE checklist was adapted from a study by Pandya et al. [5]. The chief investigator made a few modifications to the checklist to make it suitable for taking history and conducting examinations in Pediatrics. Two senior faculty members from the same department validated this modified checklist. The SCCE checklist used in the study consisted of five structured domains: history taking, general examination, systemic examination, diagnosis/treatment/complications, and communication, comprising a total of 25 items. Each item was scored using a 0-2 point scale (0 = not done, 1 = partially done, 2 = completely done), with a total possible score of 50 marks. The domain-wise weightage was as follows: history taking (15 marks); general examination (12 marks); systemic examination (13 marks); diagnosis, treatment, and complications (5 marks); and one directly observed communication skill component (5 marks) (Appendix 1). Informed written consent was obtained from all students before they enrolled in the study. Students were randomly allocated cases of different systems according to the university examination pattern by the chit-pull system. The students were allocated 45 minutes to take a history and examination following the current long-case examination pattern. On the same day, the students presented the same case in both TLCE and SCCE formats, and the assessment format to be done first was determined by picking chits. This was done to avoid any potential bias. Each assessment method was time-limited to 20 minutes, with a gap of up to a maximum of two hours between the two assessments. Students' perceptions were collected via a written questionnaire. Students were informed that the marks given for the SCCE would not be considered for their formative/internal assessment.

Outcomes

The outcome variables included mean marks, the percentage of students passing the exam, the number of students achieving good and excellent scores, and student perception, measured using a Likert scale for each assessment method.

Student feedback on both examination methods was obtained immediately after completion of the TLCE and SCCE assessments on the same day, using a structured, self-administered questionnaire. The feedback tool consisted of five close-ended items rated on a 5-point Likert scale (Strongly Disagree to Strongly Agree), with no open-ended questions. The questionnaire assessed students’ perceptions of key components, including completeness of case assessment, adequacy of weightage to history and examination, direct observation of clinical skills, importance of communication skills, and preference for future use of the examination method. Separate but identically structured forms were used for SCCE and TLCE. The complete feedback questionnaire has been provided in Appendix 2.

Assignment method and bias

Students were randomly allocated cases of different systems by the chit-pull system. They were made to present the same case in both TLCE and SCCE formats on the same day. The sequence of TLCE and SCCE was randomized for each student through a chit-pull method. Students were randomly assigned to undergo either TLCE-first followed by SCCE, or SCCE-first followed by TLCE. This randomization ensures that any learning effect would be equally likely to favor either assessment method rather than systematically biasing results in one direction. The examiners were randomly assigned to take daily TLCE or SCCE assessment duties to prevent interviewer bias.

Sample size

We employed a convenience sampling method to determine the sample size for our study, as no similar study was available to inform the calculation. In our study, 133 participants were included, and it was determined that a minimum of 40 participants was necessary to detect statistically significant differences between the two methods.

Statistical methods

Data Analysis

Marks obtained in TLCE and SCCE were compared using a paired sample t-test. Normality of TLCE and SCCE scores was confirmed using the Shapiro-Wilk test (p > 0.05 for both), supporting the use of the paired sample t-test.

Descriptive statistics were expressed as mean ± standard deviation (SD) and as frequencies with percentages, n (%). Categorical variables such as performance categories and Likert-scale responses were compared using the chi-square (χ²) test. All statistical tests were two-tailed, and a p value of <0.05 was considered statistically significant.

The statistical analysis was conducted using IBM Statistical Package for the Social Sciences Statistics software (version 21.0, IBM Corporation, Armonk, New York, USA).

## Results

**Figure 1:**
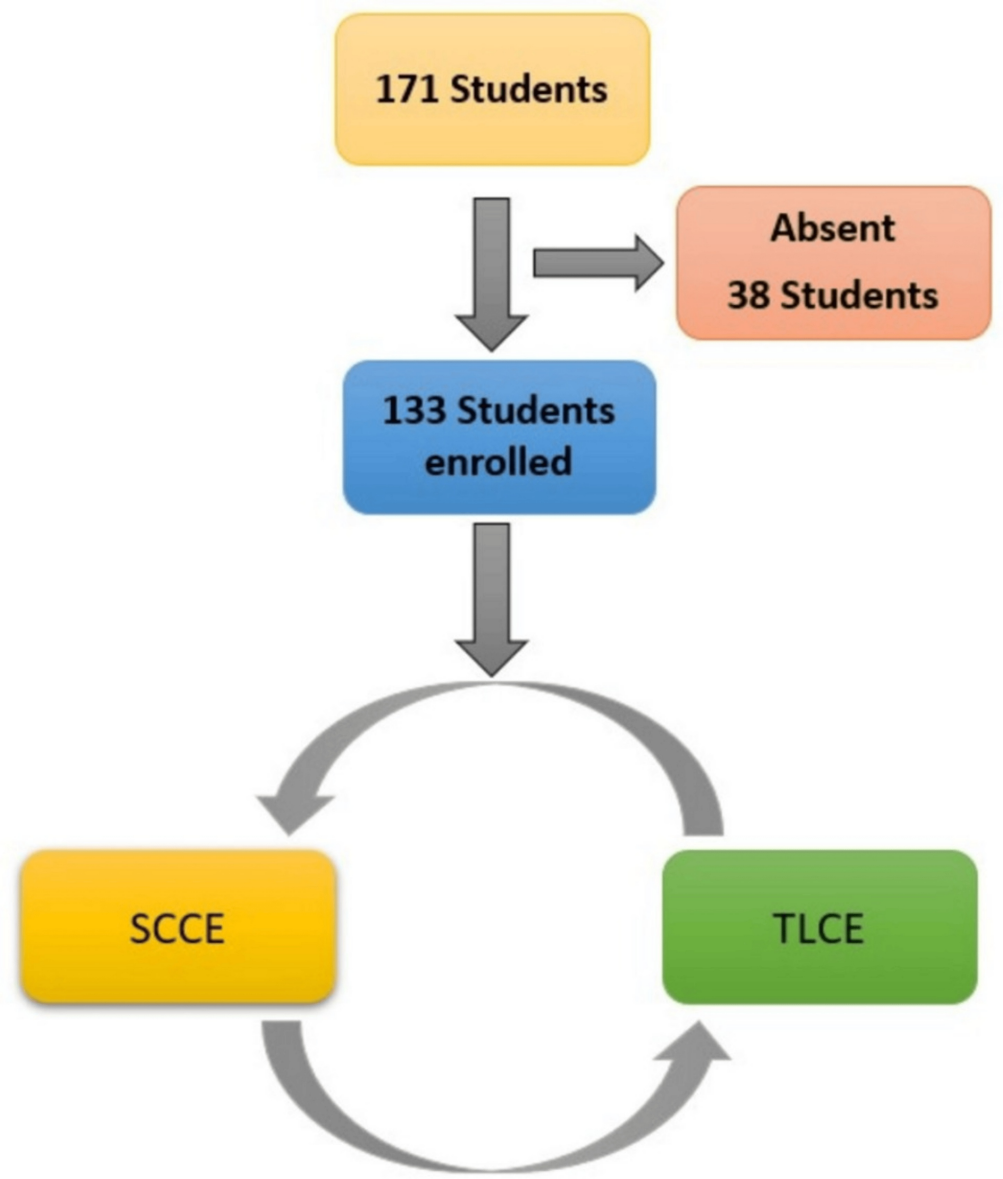
Flow diagram depicting the study process TLCE: traditional long case examination; SCCE: structured clinical case examination

A total of 133 students were enrolled in the study, comprising 74 female students (55.6%) and 59 male students (44.4%). The study flow is depicted in Figure 1.

**Figure 2:**
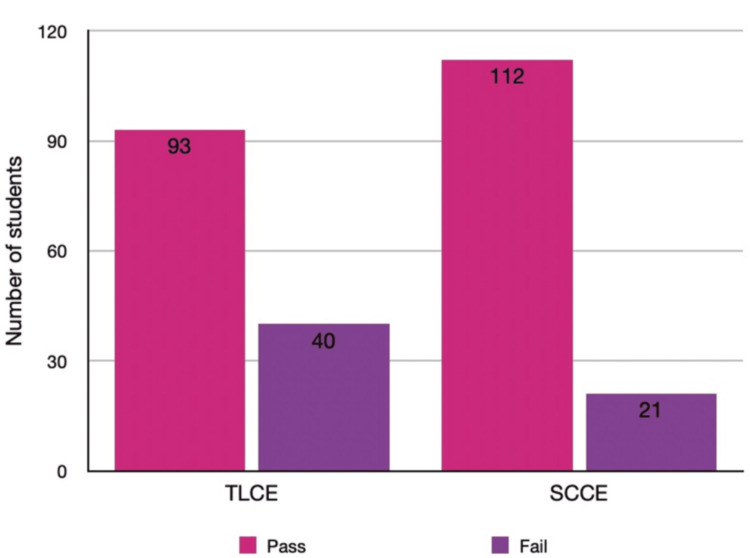
Examination result of the students of final-year MBBS in TLCE and SCCE methods of assessment Bars depict the number (n) of students who passed (≥25 marks) and failed (<25 marks) in each examination format TLCE: traditional long case examination; SCCE: structured clinical case examination

Students’ performance in the TLCE was compared with that in the SCCE. Each examination carried a maximum of 50 marks, with a passing score of 25. In the TLCE, 93 (69.9%) students scored above the passing mark, whereas in the SCCE, 112 (84.2%) did so. The mean ± SD score in TLCE was 26.7 ± 5.9, compared with 29.8 ± 5.4 in SCCE. The paired-sample t-test revealed a statistically significant improvement in SCCE performance (t = 7.21, p < 0.001). Figure 2 displays the distribution of pass and fail outcomes in both examination formats.

The marks were further stratified into four performance categories: poor (<25), average (25-29), good (30-34), and excellent (≥ 35). Table 1 shows that 40 (30.1%) students in TLCE and 21 (15.8%) in SCCE fell in the poor category. A larger proportion of students achieved good, 42 (31.6%) vs. 25 (18.8%), and excellent, 29 (21.8%) vs. 14 (10.5%), grades with SCCE compared to TLCE.

**Table 1:** Stratified marks scored by the final year MBBS students in TLCE and SCCE assessment Data are presented as n (%). Comparison between methods performed using paired sample t-test (t = 7.21, p < 0.001). p < 0.05 is considered statistically significant TLCE: traditional long case examination; SCCE: structured clinical case examination

Marks category	TLCE, n (%)	SCCE, n (%)
Poor (<25)	40 (30.1%)	21 (15.8%)
Average (25-29)	54 (40.6%)	41 (30.8%)
Good (30-34)	25 (18.8%)	42 (31.6%)
Excellent (≥35)	14 (10.5%)	29 (21.8%)
Total	133 (100%)	133 (100%)

**Figure 3:**
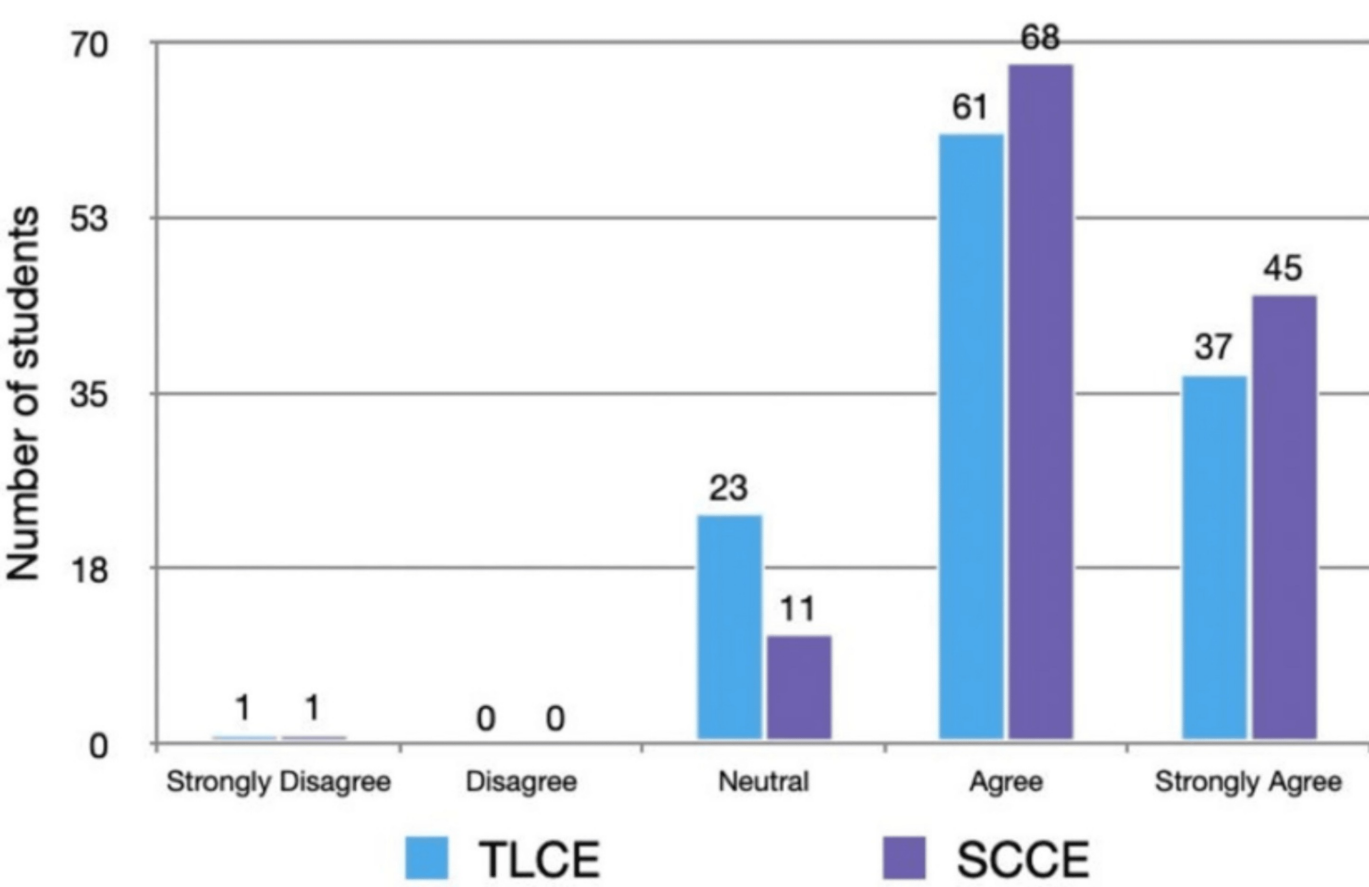
Student feedback on their perception of TLCE and SCCE method in assessing the components of clinical case presentation using Likert scale Student feedback (n) on their perception of TLCE and SCCE methods in assessing components of clinical case presentation using a Likert scale TLCE: traditional long case examination; SCCE: structured clinical case examination

Student feedback on both assessment methods was obtained using a five-point Likert scale. For the assessment of clinical-skill performance, 57 (42.9%) students agreed, and 34 (25.6%) strongly agreed that TLCE was effective, compared with 65 (48.9%) and 51 (38.3%), respectively, for SCCE. Only 1 (0.8%) student strongly disagreed with each method. A comparison of Likert-scale responses between TLCE and SCCE revealed a statistically significant difference (χ² = 13.7, p = 0.008). Table 2 summarizes the detailed distribution, showing a significant difference between the two formats (p = 0.008). Figure 3 illustrates the same data graphically.

**Table 2:** Student feedback on their perception of TLCE and SCCE method on assessment of clinical skill performance by direct observation using a Likert scale Data are presented as n (%). Group differences are analyzed using the chi-square test (χ² = 13.7, p = 0.008). p < 0.05 was considered statistically significant. Eleven students (8.3%) did not submit feedback for TLCE, and seven students (5.3%) for SCCE TLCE: traditional long case examination; SCCE: structured clinical case examination

Perception scale	TLCE, n (%)	SCCE, n (%)
Strongly disagree	1 (0.8%)	1 (0.8%)
Disagree	7 (5.3%)	0 (0%)
Neutral	23 (17.3%)	9 (6.8%)
Agree	57 (42.9%)	65 (48.9%)
Strongly agree	34 (25.6%)	51 (38.3%)
Total responses (analyzed)	122 (100%)	126 (100%)

Overall, the results indicate that SCCE yielded significantly higher mean scores and more favorable student perceptions compared to TLCE.

## Discussion

This study aimed to compare the scores obtained from the SCCE assessment method with those from the TLCE and to investigate students' perceptions of this new method. The overall scores achieved by the SCCE method were significantly higher than those of TLCE. The student feedback on perception revealed strong agreement with the SCCE method.

The study compared the performance of final-year MBBS undergraduates in assessing clinical competence using two different examination methods: TLCE and SCCE. The results indicated a significant increase in marks obtained in SCCE compared to TLCE, with 84.2% of students passing SCCE compared to 69.9% passing TLCE. The average marks obtained were also higher in SCCE (29.8 marks) than in TLCE (26.7 marks), with a significant difference observed between the two methods (p < 0.01). Additionally, a higher proportion of students scored good (31.5%) and excellent (21.8%) marks in SCCE compared to TLCE (18.7% for good, 10.5% for excellent).

Furthermore, student feedback indicated a strong preference for SCCE, with a higher percentage of students agreeing or strongly agreeing that SCCE is the most effective method for assessing both case presentation components and clinical skill performance. By interpreting our results cautiously, it can be noted that SCCE may offer advantages over TLCE in assessing clinical competence among final-year medical students. The structured nature of SCCE, combined with the use of a validated checklist, likely contributed to higher scores and more positive student perceptions. These findings align with the study's objectives, which aimed to address challenges in assessing clinical acumen and explore alternative examination methods.

The TLCE and the SCCE have distinct strengths and weaknesses in terms of their methodology, reliability, validity, feasibility, and scalability. The TLCE is highly regarded for its face validity as it evaluates the doctor's integrated clinical reaction with real patients, which mirrors real-world practice. However, intercase variability and the limited ability to cover the curriculum broadly significantly compromise its reliability [4,6]. Organizing patient encounters and conducting the assessment is a time-intensive process, which raises concerns about the feasibility of the examination. The logistical challenges involved in this process exacerbate concerns about the feasibility of the assessment [6].

Several modifications to the traditional long case have been proposed to address the issues related to its reliability. These modifications include structuring the format and marking scheme, increasing the number of examiners, and increasing the number of cases. Research suggests that improving the structure of tests and testing across more cases can enhance the reliability of the traditional long case. However, the feasibility of implementing these modifications remains a key challenge [6]. The SCCE is a medical evaluation that uses formats such as the mini-clinical evaluation exercise and the objective structured long examination record. This examination aims to maintain the benefits of the TLCE while increasing its reliability through more structured and repeated assessments [7].

When it comes to scalability, the TLCE is not as advantageous as structured examinations. This is because the former requires real patients and involves individualized assessments. On the other hand, structured examinations can be more easily scaled and standardized, although they may require considerable resources to maintain reliability at the pass/fail interface [7].

Overall, while the TLCE offers a holistic and valid assessment of clinical competence, its reliability and feasibility are major limitations. SCCE attempts to balance the need for a valid evaluation with a more reliable, feasible, and scalable method, but they also require careful design and resource allocation to achieve these goals [4,6,7].

The choice between TLCE and SCCE has implications for the design of medical education curricula, teaching methodologies, and student learning outcomes. The SCCE format allows for a broader sampling of the curriculum and a more objective assessment of clinical competencies [8]. Curriculum reforms that include structured clinical teaching and assessment methods have been shown to improve students' performance in history taking and physical examination skills [9].

In India, the medical education system has undergone a significant change in the last decade, with a revision of the undergraduate curriculum and a shift from knowledge-based medical education to competency-based medical education (CBME) [10].

CBME aims to produce competent doctors who possess proficient knowledge and good clinical and communication skills. Regular and comprehensive evaluation of competencies plays a vital role in creating competent and responsible healthcare professionals. However, the CBME model presents certain challenges, including the need for frequent and objective assessments, which necessitate considerable faculty involvement and resources. Poor standardization of patients and examiners, assessment by only a limited number of examiners on fewer cases, and a lack of systematic feedback from students and examiners can decrease the reliability and validity of assessments [10]. SCCE has demonstrated its effectiveness in addressing these drawbacks [11]. As a result, it can be utilized as an assessment instrument for CBME.

Although SCCE produced higher scores and more favorable student perceptions, these findings should be interpreted cautiously. The differences may reflect the structured, checklist-based scoring system rather than true variations in clinical competence, and the present study did not evaluate whether SCCE can discriminate effectively between high- and low-performing students. Therefore, the results do not establish SCCE as a superior assessment tool, and further studies are needed to assess its discriminative and predictive validity.

Comparison with previous studies

The SCCE has been compared to the TLCE to assess clinical competence. Studies have shown that SCCE better categorizes students into competent, average competent, and noncompetent groups, while TLCE tends to cluster students in the average group [5]. The SCCE evaluation system is better than TLCE, as it helps identify areas of weakness and covers a wide range of clinical skills [12]. The main disadvantage of TLCE is its inability to sample the curriculum widely, resulting in low reliability, while its advantage lies in assessing the candidate's overall approach to the patient [13]. Critics have suggested improving the TLCE rather than abandoning it, and modifications have been proposed to increase reliability and maintain the holistic approach [3]. The objective structured clinical examination has also been evaluated and found to be a reliable and valid test of clinical skills, with correlations to traditional assessments [14].

Considering the diversity of analyses and the positive results from similar studies, the findings of this study suggest that SCCE holds promise as a comprehensive and effective method for assessing clinical competence in medical education. However, a cautious interpretation is warranted, and further research in diverse settings is needed to confirm these findings and address the identified limitations.

Limitations

This study has several limitations. It was conducted in a single medical school, which may limit generalizability to institutions with different settings and resources. As the study was restricted to pediatrics, the relative effectiveness of SCCE vs. TLCE may differ across other medical specialties. Although student feedback was obtained, perceptions of other stakeholders, such as faculty and clinical supervisors, were not included. Information sharing between students who completed the examinations earlier and those assessed later may have influenced performance. The use of multiple examiners could also introduce subjective bias. Another key limitation is the potential practice or learning effect, as students assessed the same patient in both formats on the same day. Despite randomizing the order of TLCE and SCCE, repeated exposure to the same clinical scenario may have improved performance in the second examination, partially inflating score differences.

Generalizability

While the study results demonstrate the potential benefits of SCCE in assessing clinical competence in pediatrics, health educators should critically evaluate the applicability of these findings to their specific contexts. Consideration of contextual factors, validation of assessment tools, and adaptation of educational practices are essential for enhancing the generalizability and relevance of the study results to health educators worldwide.

Suggestions

Similar studies with larger sample sizes across multiple medical colleges and departments could provide insights for shaping future examination patterns.

## Conclusions

In conclusion, the findings of this study suggest that SCCE may offer certain advantages over TLCE in providing a more structured and student-perceived favorable format for assessing clinical competence among final-year medical students. The higher scores achieved with SCCE, together with strong student agreement, indicate its potential to address some challenges associated with traditional assessment methods; however, these results should be interpreted with caution, as the score differences may partly reflect the structured checklist rather than true differences in competence. Nonetheless, the comparison with previous studies indicates a consistent trend favoring SCCE's ability to categorize students more effectively and assess a wider range of clinical skills.

## Appendices

Appendix 1

**Table 3:** SCCE evaluation sheet SCCE evaluation checklist used for assessing final-year MBBS students. The checklist consists of five domains: history taking, general examination, systemic examination, diagnosis/treatment/complications, and communication skills, comprising 25 items with a total score of 50 marks. Each item is scored on a structured scale from 0 to 2 based on completeness and accuracy of performance SCCE: Structured Clinical Case Examination

Sl. no.	Evaluation points	Total marks	Marks obtained
(A) History taking			
1	Whether demographic data taken?	1	
2	Chief complaints: all symptoms elaborated and chronology maintained	2	
3	Origin, duration, progress: a) All symptoms elaborated in detail b) Minute points noted	4	
4	Relevant past and family history, birth, development, diet, and immunization history	3	
5	Direct observation: Detailed symptom history OR detailed past/family/development/diet history	5	
Subtotal		15	
(B) General examination			
1	Vital data: All parameters properly taken	1	
2	Anthropometry and interpretation	1	
3	Head-to-toe examination done	1	
4	All positive findings elicited	1	
5	Direct observation: demonstration of one general examination sign	5	
6	Direct questions related to general examination	3	
Subtotal		12	
(C) Systemic examination			
1	Systemic examination done in proper proforma	2	
2	Able to find positive systemic signs	2	
3	Relevant other system findings	1	
4	Direct observation: demonstration of a systemic sign	5	
5	Direct questions related to systemic examination	3	
Subtotal		13	
(D) Diagnosis, treatment, complications			
1	Able to reach a clinical diagnosis/differential diagnosis	3	
2	Investigations/complications	1	
3	Treatment options	1	
Subtotal		5	
(E) Communication skill - direct observation			
1	Explain prognosis/nature of the disease to patient/parent	5	
Subtotal		5	
Grand total		50	

Appendix 2

**Table 4:** Student feedback questionnaire used for evaluating perceptions of the SCCE and the TLCE SCCE: Structured Clinical Case Examination; TLCE: traditional long case examination

Sl. no.	Question	Strongly disagree	Disagree	Neutral	Agree	Strongly agree
Feedback of students - structured clinical long case examination						
1	Does SCCE assess all components of case presentation?					
2	Were all components of history, examination, clerking, and counseling given appropriate weightage in SCCE?					
3	Is the assessment of clinical skill performance by direct observation done in SCCE?					
4	Is assessment of communication/counseling skills important to evaluate in practical examination?					
5	Whether we should use this method of SCCE in case evaluation for students?					
Feedback of students - traditional clinical case examination						
1	Does TLCE assesses all components of case presentation?					
2	Were all components of history, examination, clerking, and counseling given appropriate weightage in TLCE?					
3	Is the assessment of clinical skill performance by direct observation done in TLCE?					
4	Is assessment of communication/counseling skills important to evaluate in practical examination?					
5	Whether we should use this method of TLCE in case evaluation for students?					
